# Genetic modification of cystic fibrosis with ΔF508 mutation of CFTR gene using the CRISPR system in peripheral blood mononuclear cells

**DOI:** 10.22038/ijbms.2020.50051.11415

**Published:** 2021-01

**Authors:** Sepideh Khatibi, Mohammadreza Modaresi, Reza Kazemi Oskuee, Mohammad Salehi, Seyed Hamid Aghaee-Bakhtiari

**Affiliations:** 1 Department of Medical Biotechnology and Nanotechnology, Faculty of Medicine, Mashhad University of Medical Sciences, Mashhad, Iran; 2 Division of Pediatrics Pulmonary Disease, Children’s Medical Center, Pediatrics Center of Excellence, Tehran University of Medical Sciences, Tehran, Iran; 3 Department of Biotechnology, School of Advanced Technologies in Medicine, Shahid Beheshti University of Medical Sciences, Tehran, Iran; 4 Bioinformatics Research Group, Mashhad University of Medical Sciences, Mashhad, Iran

**Keywords:** CFTR gene, CRISPR, Cystic fibrosis, Peripheral blood - mononuclear cells, ΔF508 mutation

## Abstract

**Objective(s):**

Cystic fibrosis (CF) is an inherited autosomal recessive disease that is caused by mutations in the cystic fibrosis transmembrane conductance regulator (CFTR) gene. The present study aimed to investigate the genetic modification of CF with ΔF508 mutation of the CFTR gene using CRISPR in peripheral blood mononuclear cells (PBMCs).

**Materials and Methods:**

Two single guide RNAs were designed to target sequences in the CFTR gene. The transfection efficiency of PBMC cells was examined through evaluation of green fluorescent protein (GFP) expression using fluorescent microscopy. Moreover, a sgRNA-Cas9 plasmid was tested to target the CFTR gene. The ΔF508 gene modification was evaluated and confirmed by PCR and Sanger sequencing methods.

**Results:**

Our results indicate the feasibility of site-specific gene targeting with the CRISPR/Cas9 system. 33% of the samples were corrected using CRISPR in mutant locus and confirmed by sequence blast at NCBI databases and primers outside the arm locus. CRISPR/Cas9 approach represents an efficient tool to repair the ΔF508 mutation of the CFTR gene in PBMC Cells.

**Conclusion:**

Therefore, the CRISPR system can be highly efficient and specific and provides a powerful approach for genetic engineering of cells and model animals. Generally, the proposed method opens new insights into the treatment of human diseases.

## Introduction

Cystic fibrosis (CF) is a multi-system autosomal recessive disorder, including lung injury, pancreatic insufficiency, liver disease, intestinal dysfunction, and high concentrations of chloride in the sweat. The cause of the disorder is mutation in the cystic fibrosis transmembrane conduction regulator gene (CFTR) (1-3). The CFTR gene is located on the long arm of chromosome 7 (7q31.2) and encodes the ion chloride channel. CFTR protein is found at the cell membrane surface of a wide spectrum of tissues and mostly acts as a chloride ion channel, which is regulated by cAMP-dependent phosphorylation (2).

A study found unusual DNA repeats with unknown functions in the genomic DNA of *Escherichia coli* (4). Thereafter, Mojica *et al.* identified similar sequences in other bacteria and called them Clustered Regularly Interspaced Short Palindromic Repeats (CRISPR) (5-7). Finally, the CRISPR sequences were introduced as an immune system in bacteria, which consists of two elements: repeat segments that have the same composition in the given bacteria, and the spacers of similar length that are unique and represent the segments of foreign nucleic acids. Transcription of the CRISPR region produces short RNA molecules, crRNA, which contain sequences complementary to previously encountered foreign nucleic acids. These crRNA then direct CRISPR-associated (Cas) proteins to find and destroy the complementary target sequences on invading viruses’ DNA molecules by cutting them in pieces (8, 9). In this process, the Cas protein act as the targeting means which is used by scientists to find and break a specific DNA molecule in genomic DNA. The special feature of Cas nuclease makes it possible to edit the genome of live cells by replacing the RNA in the CRISPR system with a targeted RNA molecule and transferring it back to the cell (10). So far, Cas9 nucleases from *E. coli* have been studied and are used more than other identified Cas nucleases in other bacterias (11).

Several studies investigated the use of the CRISPR/Cas9 system for human diseases such as Duchenne Muscular Dystrophy (12), CF (13), Barth syndrome (14), β-thalassemia (15), and hemophilia (16). The diseases are caused by single-nucleotide mutations and the CRISPR/Cas9 system was used to correct the mutation in cellular models. Also, scientists have used CRISPR/Cas9 for the treatment of some human diseases in animal models, such as the mouse model of tyrosinemia (17), lung cancer (18), and monkey model of muscular dystrophy (19). The correction of genetic diseases by the CRISPR system in human embryos or embryonic animal models has not been well studied. Recently, a study (2017) by modifying the mutations in HBB and G6PD genes in human embryos, showed that the CRISPR/Cas9 system is highly effective in genetic modification of human embryos (20). Also, Liang *et al.* (2015) used the CRISPR/Cas9 system to modify β thalassemia in the human embryo (21). However, there is very limited information on the performance of the CRISPR system in genetic modification of embryos. Therefore, this study aimed to evaluate the genetic modification of CF mutation using the CRISPR system in Peripheral Blood Mononuclear Cells (PBMCs).

## Materials and Methods

***gRNA design and preparation***

In the present study, the gRNA design was performed according to the Schwank *et al.* defined method (13). sgRNA sequences were ordered in the form of phosphorylated forward and reverse oligonucleotides flanked by desired restriction sites. The sequences of used gRNAs were as follows: gRNA1: ACCATTAAAGAAATATCAT. gRNA2: ATGCTTTAAGAACCTTGCAA.

***gRNA and Cas9 expressing vector***

In this study, the CRISPR expressing gRNA-GFP-X330 vector was used to express codon and sgRNA. Antibiotic resistance genes used as the selection markers were ampicillin in the pX330 vector. In order to target sequences in the CFTR gene, the gRNA-GFP-X330 vector was constructed. To clone a gRNA into a gRNA-GFP-X330 vector, the vector was digested with BbsI enzyme, and then both vectors were treated with Alkaline Phosphatase. Complementary oligos of CFTR was heated at 95 °C for 5 min and annealed by decreasing the temperature from 0.5 °C/s to 22 °C using a thermocycler. Then, the short double-strand DNA fragments were ligated into the linearized BbsI site of gRNA-GFP-X330.

***Ligation confirmation***

In order to confirm ligation and correct direction of inserts, digestion with restriction enzymes and sequencing were performed. For the restriction enzyme digestion, the cloned gRNA-GFP-X330 vector (in which the BbsI restriction site has been removed after cloning) was digested with the BbsI restriction enzyme. For the sequencing, desired primers for regions surrounding the target sites in the CFTR gene were designed. The used primers were as follows: sg-Forward: CACGACAGGTTTCCCGACT, sg1-Reverse: CACCGACCATTAAAGAAAATATCATG, and sg2-Reverse: CGTTGCAAGCTTCTTAAAGCAT. The sequencing results were analyzed by sequencing to confirm the correct direction of inserts (Pishgam, Iran).

***CRISPR vector transfection***

The human embryonic kidney cells (HEK293, ATCC CRL-1573) were purchased from Stem Cell Technology Research Center, Tehran, Iran. The HEK293 cell culture was performed using RPMI 1640 medium supplemented with 10% fetal bovine serum (FBS) and 1% penicillin-streptomycin (100 units/ml-100 µg/ml) antibiotics (Gibco, United States America), and incubated under standard conditions at 37 °C and 5% CO_2 _(Memmert, Germany). The cells were seeded in a 96-well plate (1.5 × 10^4^ cells/well) and incubated for 24 hr under standard conditions. Then, HEK293 cells were transiently co-transfected with the Cas9-gRNA vector using Lipofectamine 2000. Finally, the rate of transfection was assessed by fluorescent microscopy after 24 hr.

***Confirmation of enzyme Cas9***

The genomic DNA of HEK293 cells was extracted using a DNA Extraction Kit (iNtRON Biotechnology, South Korea) and was amplified using PCR using the following primers: first pair primer Forward: AATGATGATTATGGGAGAACTGGAG and Reverse: TTGGGTAGTGTGAAGGGTTCATATG; second pair primer Forward: ACCATTAAAGAAATATCAT and Reverse: TTGGGTAGTGTGAAGGGTTCATATG). The thermocycler conditions were as follow: initial denaturation (1 cycle at 94 °C for 3 min), denaturation (35 cycles at 94 °C for 30 sec), annealing (35 cycles at 52-60 °C for 30 sec), extension (35 cycles at 72 °C for 60 sec), and final extension (1 cycle at 72 °C for 5 min). The PCR products were electrophoresed using 2% agarose gel stained by ethidium bromide.

***Isolation and culture of PBMCs***

In the current study, 3 patients with CT (Δ508 mutation on chromosome 7) were recruited from Children’s Medical Educational Hospital, Tehran, Iran. The peripheral blood (5 mL) was drawn from all participants and collected into vials containing heparin (2 μl) as an anticoagulant. Then, the blood samples were diluted using RPMI medium (5 ml). The diluted blood samples were added to a sterile falcon containing Ficoll medium (5 ml) and then centrifuged (4000 rpm) at 18 °C for 30 min. The second formed layer was considered as PBMCs. The obtained PBMCs were suspended with RPMI medium (5 ml) and then centrifuged (1500) rpm at 18 °C for 30 min. Then, obtained cellular sediment was resuspended by RPMI medium and transferred to a cell culture flask containing RPMI supplemented by 10% FBS. After 24 hr, the supernatant medium containing lymphocytes cells was cultured in another cell culture flask with complete RPMI under standard conditions. Moreover, the adhered cells layer containing monocyte cells were cultured in other cell culture flasks with complete RPMI under standard conditions.

***Transfection of PBMCs by lipofectamine and PolyFect***

The PBMCs were seeded in a 24-well plate (1.5 × 10^4^ cells per well) and incubated for 24 hr under standard conditions. After overnight culture, transfection of PBMCs was performed using PolyFect, Lipofectamine 2000, and Lipofectamine 3000 agents. The RPMI medium (1 ml) and Lipofectamine 2000 or 3000 agents (1 μl) containing the PMAX-GFP vector were added in two wells. Also, a well without any transfection agents and vector was considered as control. After 17 hr, the transfected cells were washed with complete RPMI medium to remove Lipofectamine agents. Moreover, RPMI medium (FBS-free and antibiotics-free) (25 μl) containing PMAX-GFP vector (0.3 μg) and PolyFect, agent (2.5 μl) was added in two other wells. Ultimately, the cell transfection rate was evaluated by fluorescence microscope.

***PBMCs transfection and gene modification***

The transfection of PBMCs was performed using the PMAX-GFP vector and PolyFect agent (as the best carrier of the CRISPR vectors) in presence of GFP. The cell transfection rate by the PolyFect agent was evaluated by a fluorescence microscope after 48, 72, and 120 hr. After 120 hr, the puromycin antibiotic (1 μg/ml) was used to select the transfected cells. Finally, the genomic DNA of selected cells was extracted using a DNA extraction kit (iNtRON Biotechnology, South Korea) according to the manufacturer’s protocol. Then, DNA samples of PBMCs were amplified using specific primers under the following conditions: initial denaturation (1 cycle at 95 °C for 5 min), denaturation (35 cycles at 94 °C for 30 sec), annealing (35 cycles at 60 °C for 30 sec), extension (35 cycles at 72 °C for 60 sec), and final extension (1 cycle at 72 °C for 1 min). The PCR products were electrophoresed using 2% agarose gel stained by ethidium bromide. Moreover, the obtained PCR products were further analyzed using the Sanger sequencing method (Macrogen, South Korea).

***Duplicate regions outside of ARM sequence***

**Figure 1:**
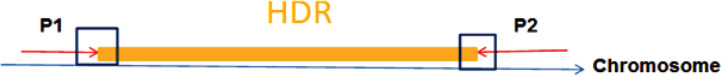
The location of upstream and downstream primers of the HDR region on chromosome 7 which showed the overlapping regions

To verify the modified sequence in the DNA of transfected cells, the 300 bp downstream region homology-directed repair (HDR) sequence was amplified by designed primers (Figure 1). The used primers were the following sequences: Forward: CCCCATTCCAGCTTTAAACA and Reverse: CCACAAATTCCCGAAGTATATGA). Moreover, the obtained PCR products were further analyzed using the Sanger sequencing method (Macrogen, South Korea).

## Results

***Confirmation of gRNA1 and gRNA2 presence in vector***

In the present study, we targeted a region in the CFTR gene in which two gRNA1 and gRNA2 were separated by 550 bp and 280 bp on 2% agarose gel, respectively. 

***Confirmation of Cas9 enzyme cutting***

**Figure 2:**
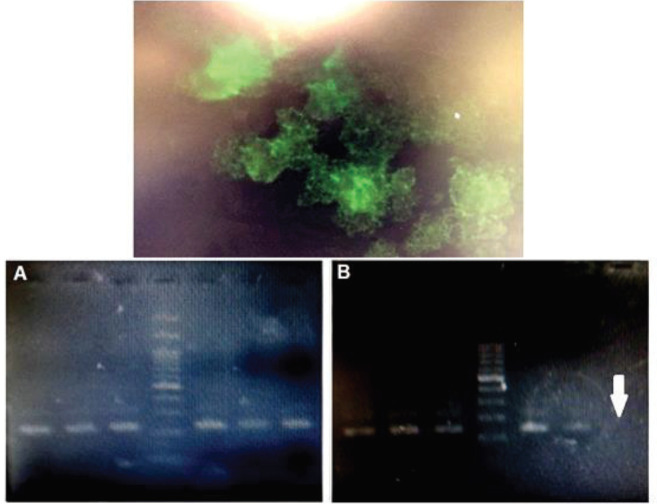
Electrophoresis of PCR products on 2% agarose gel in order to find the ΔF508 gene-modified HEK293 cells. (A) The first step product was a 237 bp fragment. (B) The second step product was a 190 bp fragment. No band formation indicates cutting by Cas9

In HEK293 cells, the rate of transfection of CRISPR vectors was visualized about 50% after 24 hr by Lipofectamine 2000 based on fluorescent microscopy (Figure 1). To evaluate of ΔF508 gene modification in HEK293 cells, the PCR products were electrophoresed on 2% gel agarose in order to find the ΔF508 gene-modified HEK293 cells. To select the HEK293 clonal populations gene modification was performed, a two-step RCR was used. The first step amplified a 237 bp fragment. The first-round PCR product was used as the template for second-round PCR and the second step amplified a 190 bp fragment (Figure 2).

***CRISPR vector transfection in HEK293 cell line and PBMCs***

**Figure 3:**
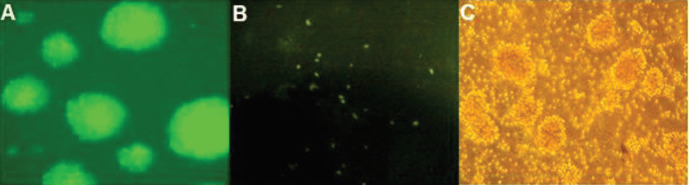
Transfection rate of HEK293 and PBMCs. (A) HEK293 cell transfection with CRISPR vectors. (B) PBMCs before transfection (left), efficiency of PBMCs transfection was about 50%, after 72 hr (middle), efficiency of PBMCs transfection was about 80%, after 120 hr (right)

HEK293: human embryonic kidney cells; PBMCs: peripheral blood mononuclear cells; CRISPR: clustered regularly interspaced short palindromic repeats

In PBMCs cells, the cells were distinguished in green colonies due to the presence of phytohemagglutinin PHA and were observed by fluorescence microscopy after 72 and 120 hr. After 24, 72, and even 120 hr, the PBMCs were transfected about 2–3% with Lipopactamine 2000 and Lipofectamine 3000. After 24 hr, PBMCs were transfected about 2–3% by PolyFect. However, after 72 hr, PBMCs were transfected about 50%. Also, after 120 hr, the transfection rate of these cells increased by about 80%. Transfection results showed that PolyFect could be the best carrier of transfection in PBMC cells (Figure 3B).

***Confirmation of PBMCs transfection by flow cytometry***

**Figure 4:**
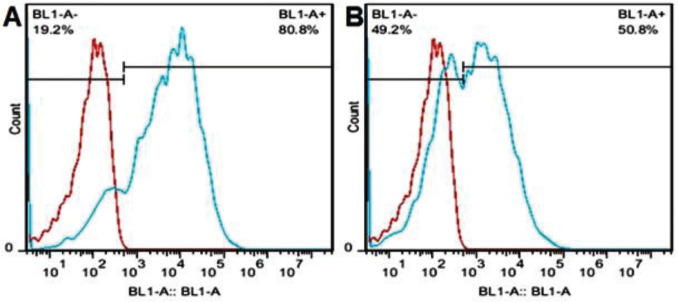
Transfection rate of PBMC cells using PolyFect after 72 (A) and 120 (B) hr was 50 and 80%, respectively

According to the flow cytometry method, transfected cells with PolyFect and lipoctamine 2000 and 300 were 2% after 24 hr; whereas transfected cells with PolyFect and PMAX-GFP vector were 50% after 72 hr and 80% after 120 hr (Figure 4).

***Mutation correction in PBMCs***

**Figure 5:**
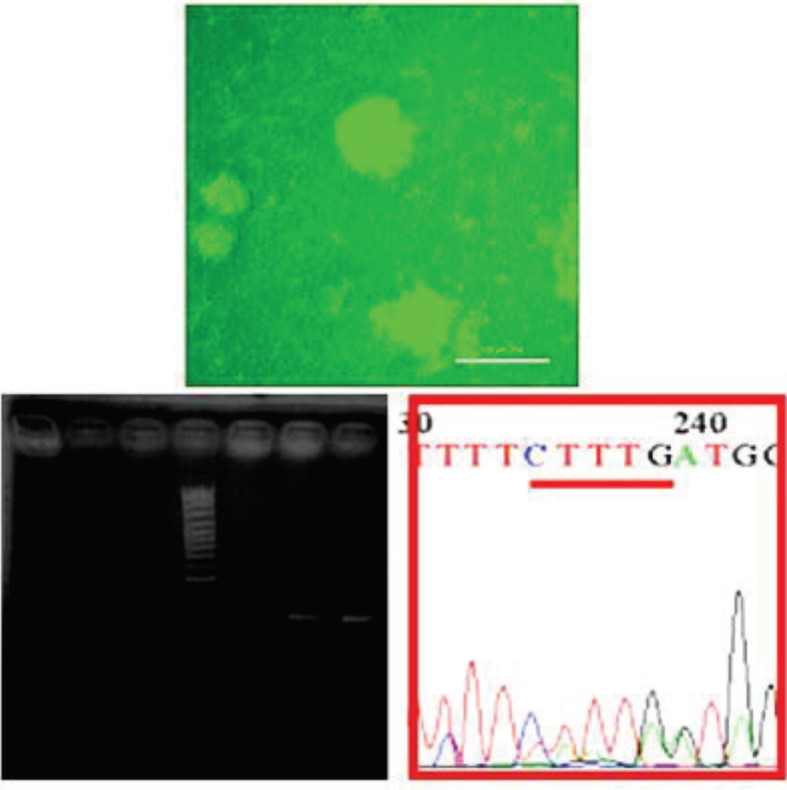
PBMC isolated from a transfected patient by CRISPR after 120 hr. The modified sequence is shown in the graph

PBMC: peripheral blood mononuclear cells; CRISPR: clustered regularly interspaced short palindromic repeats

**Figure 6:**
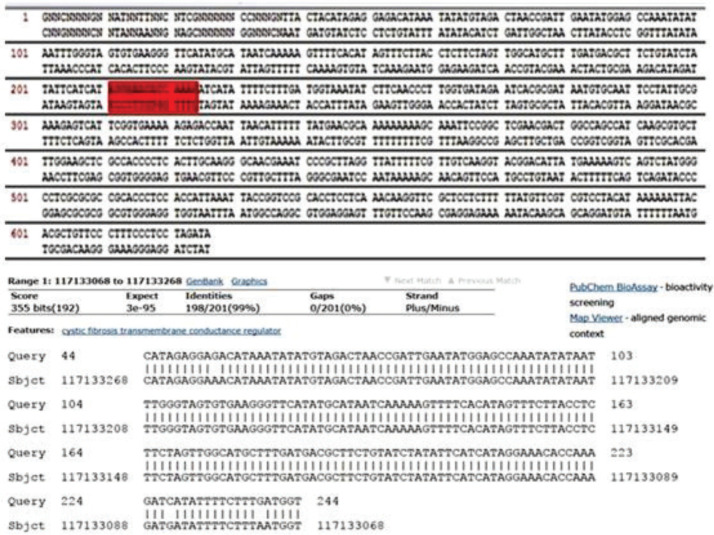
The blast PCR modified product

The PBMCs isolated from the patients were transfected using PolyFect by CRISPR and 60% of the cells were transfected. The Δ508 mutation was amplified using a specific primer. Among the three PBMCs samples, only one showed a correction of the ctt mutation site. Therefore, according to our findings, 33% of the samples were corrected at the ctt deletion site (Figures 5 and 6, Table 1).

***Evaluation of gene editing by outside ARMS ragin***

**Figure 7:**
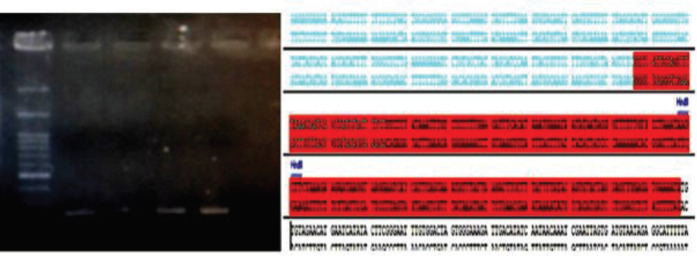
Specimens amplified a 250 bp product with the HDR downstream primer at temperatures of 55–58 °C. The green sequence represents the HDR sequence, and the red sequence represents the sequence obtained from the amplification of the HDR region downstream, which overlapped the 38 bp with the HDR region. Therefore, the transfected cell modifications were confirmed

The PCR products obtained from the extraction were analyzed by the Sanger sequencing method and the results were aligned with the results at the NCBI site (https//www.ncbi.nlm.nih.gov/gene). The results showed that one of the three samples with Δ508 mutations was modified in the ctttt sequence. Also, in the sequence graph of this sample, the deletion cttt was modified. In addition to the specific primer, another primer outside the ARMS region in the upstream of the mutation region was designed, amplification of which indicated that the modified region was on genomic DNA and confirmed the modified sequence. Specimens amplified a 250 bp product with the HDR downstream primer at temperatures of 55–58 °C. Therefore, amplified downstream samples were sequenced for further investigation, and the results indicated a 38 bp overlapping with the downstream of the HDR region (Figure 7).

HDR: homology-directed repair

PBMC: peripheral blood mononuclear cells

HDR: homology-directed repair

**Table 1:** Peripheral blood mononuclear cells (PBMCs) samples clustered regularly interspaced short palindromic repeats (CRISPER) transfection with polyfact and modified samples rate

**Modified samples**	**CRISPER transfection with polyfact (%)**	**Derived cells**	**Blood samples**
33%	60%	PBMC	3

## Discussion

CF is caused by mutations in the CFTR gene that lead to defective chloride and bicarbonate transport and dysregulation of sodium transport via loss of epithelium sodium channel (ENaC) inhibition by the CFTR protein. The most serious manifestation of CF is reduced mucociliary clearance in the lung. This results in the development of viscous hypoxic mucus which promotes recurrent *Pseudomonas aeruginosa* infections and concomitant loss of lung function. Damage to the lung architecture occurs from both *P. aeruginosa* virulence factors and increased neutrophil-mediated necrosis resulting in inflammation (1-3). CF presents as a target for novel therapeutics due to reports that low-level correction of CFTR transcript levels (5–10%) or of defective cells (25%) (22) may be sufficient to restore a normal phenotype.

PBMCs are the main components of the immune system and can be isolated from peripheral blood. These cells are very important in the study of drug toxicity or new chemical compounds. Also, the genetic manipulation of these cells is essential for basic studies in immunology and applied medical research. The aim of gene transfer to PBMCs can be different, including induction of the immune system, gene therapy, and creation of unique features (23). So far, various methods with disadvantages and advantages have been developed to gene transfer to PBMC cells (24, 25). The non-viral gene transfer methods are of great importance because of high bio-security. However, they have lower efficiency compared to viral methods (26). According to the importance of gene transfer in PBMC cells, as well as the importance of non-viral gene transfer methods and low efficiency of these methods, we evaluated the efficiency of the PolyFect kit in transfection of PBMCs using the PMAX-GFP vector in comparison with Lipofectamine 2000 and Lipofectamine 3000. The levels of PBMCs transfection by Lipopactamine 2000 and Lipofectamine 3000 was observed at about 2–3%. Also, the transfection rate of these cells was calculated using PolyFect at about 70%. In a study, the transfection rate of human hematopoietic stem cells using PolyFect was reported as about 11–13% (27). In another study, the transfection rate of PBMCs by lipofectam was reported at 7.3% (28). In a study, the transfection rate of bone marrow stromal cells (BMSC) using lipofectam was observed at 11% (29). In another study, transfection rates of murine hepatoma cells, rat bone marrow mesenchymal stem cells, human synovium-derived mesenchymal stem cells, and mouse bone marrow mesenchymal stem cells (C57) using PolyFect were reported as 50%, 40%, 21%, and 10%, respectively (30). Differences in the transfection rate efficiency in the mentioned studies can be due to differences in the studied cells as well as different gene transfer conditions.

Researchers (2015) corrected 23% of lung epithelial cells using a CRISPR system (31). In another study, Schwank *et al.* (2013) corrected 14–25% of the organoid cells with 508 delta using a CRISPR system. The peripheral blood of the sample is accessible, so this method could be a practical method for studies in genetic modification (13). In summary, although given its multi-organ involvement CF does not appear to be a prime candidate for clinical application of adult stem cell gene therapy, this approach may present a safe complement to induced-pluripotent-stem-cell-based approaches, and in the future, it could be applied to different single-gene hereditary defects. The advantage of combining HR-based gene correction strategies with organoid culture technology rests in the possibility of clonal expansion of single adult patient stem cells and the selection of recombinant clonal organoid cultures harboring the desired, exact genetic change.

## Conclusion

It can be concluded that PBMC cell transfection efficiency is considerably higher by polyfactant solution at 72 and 120 hr compared to Lipofectamine 2000 and Lipofectamine 3000 with a 2–3% efficiency. Also, the transfection efficiency of PolyFect increased significantly with increasing time, in which the highest transfection efficiency was observed at 120 hr; whereas the transfection efficiency of these cells was about 2–3% by all three kits in 24 hr. In general, the results of the present study showed that the transfection efficiency of PBMCs by PolyFect at higher times was more than Lipofectamine 2000 and Lipofectamine 3000. However, different results may be obtained from studies on other cells, due to the physiological differences of different cells. To achieve the highest transfection efficiency of PBMC cells, further studies on the transfection of these cells and other cells are recommended by various methods. 
